# CoO_*x*_ nanoparticle anchored on sulfonated-graphite as efficient water oxidation catalyst†
†Electronic supplementary information (ESI) available. See DOI: 10.1039/c7sc01756a



**DOI:** 10.1039/c7sc01756a

**Published:** 2017-06-26

**Authors:** Jingqi Guan, Chunmei Ding, Ruotian Chen, Baokun Huang, Xianwen Zhang, Fengtao Fan, Fuxiang Zhang, Can Li

**Affiliations:** a State Key Laboratory of Catalysis , iChEM , Dalian Institute of Chemical Physics , Chinese Academy of Sciences , Dalian National Laboratory for Clean Energy , Dalian , 116023 , China . Email: fxzhang@dicp.ac.cn ; Email: canli@dicp.ac.cn

## Abstract

Development of efficient, robust and earth-abundant water oxidation catalysts (WOCs) is extremely desirable for water splitting by electrolysis or photocatalysis. Herein, we report cobalt oxide nanoparticles anchored on the surface of sulfonated graphite (denoted as “CoO_*x*_@G-Ph-SN”) to exhibit unexpectedly efficient water oxidation activity with a turnover frequency (TOF) of 1.2 s^–1^; two or three orders of magnitude higher than most cobalt-based oxide WOCs reported so far. The CoO_*x*_@G-Ph-SN nanocomposite can be easily prepared by a soft hydrothermal route to have an average CoO_*x*_ size below 2 nm. Additionally, the loading of CoO_*x*_@G-Ph-SN catalyst on the surface of a BiVO_4_ or Fe_2_O_3_ photoanode can boost remarkably the photoanode currents for robust photocatalytic water oxidation under visible light irradiation. Its excellent activity and photochemical stability for water oxidation suggest that this ultrasmall cobalt-based composite is a promising candidate for solar fuel production.

## Abstract 2

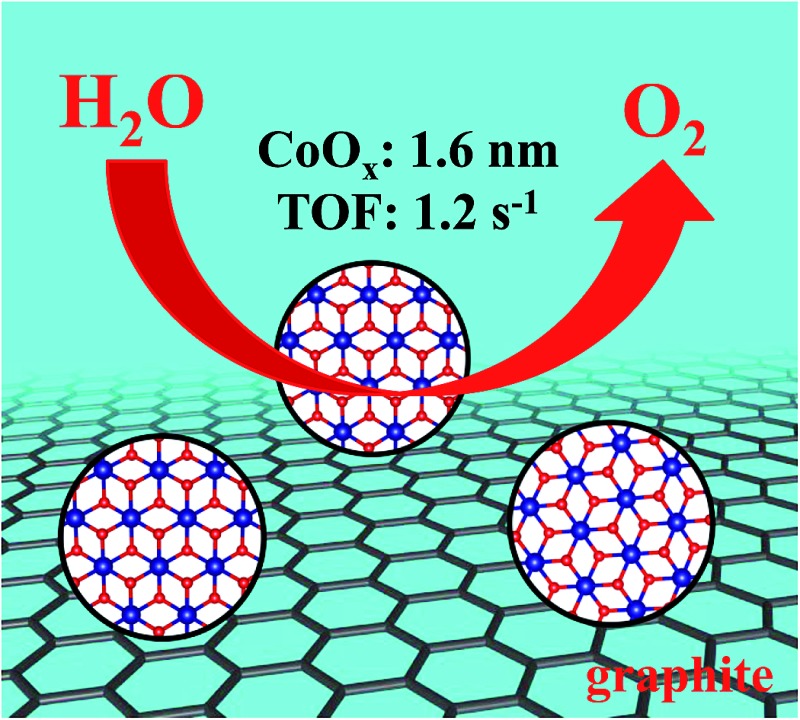
Ultrasmall CoO_*x*_ nanoparticles on sulfonated graphite exhibit highly efficient water oxidation activity and can be used for electrochemical and solar water oxidation.

## Introduction

Current energy resources are derived mainly from fossil fuels (oil, coal and gas), and the utilization of these has caused numerous environmental problems, such as the increasing emission of greenhouse gas, most notably carbon dioxide (CO_2_).^1^ To address this issue, an increased amount of clean, renewable and sustainable energy resources should be presented for energy resource distribution.^2^ Solar energy is the largest clean and renewable energy source on the Earth.^3^ Solar-driven water splitting can store energy in the form of hydrogen fuel,^2^ and water oxidation is considered to be the key reaction for overall water splitting.^4,5^ Therefore, the development of highly efficient and robust water oxidation catalysts (WOCs) is crucial to the implementation of solar fuel production.

In the past decades, continuous efforts have been devoted to developing efficient and robust WOCs. Mn-,^6–8^ Fe-,^9^ Co-,^10–13^ Ni-,^14^ Ru-,^15–17^ and Ir-based^18^ complexes or metal oxides have been examined for water oxidation by chemical, electrochemical or photocatalytic approaches. Some homogeneous molecular complexes, such as [Ru(bda)(isoq)_2_] (H_2_bda = 2,2′-bipyridine-6,6′-dicarboxylic acid; isoq = isoquinoline)^15^ and halogen substituted [Ru(bda)(isoq)_2_]^17^ exhibited comparable catalytic activity for water oxidation to the CaMn_4_O_5_ complex in PSII of natural photosynthesis. In addition, cobalt-based polyoxometalates [Co_4_(H_2_O)_2_(PW_9_O_34_)_2_]^10–^ and Na_10_[Co_4_(H_2_O)_2_(VW_9_O_34_)_2_]·35H_2_O showed very high catalytic activity (TOF = 5 s^–1^) for water oxidation in the [Ru(bpy)_3_]^2+^-sulfate system under light irradiation.^11,13^ Although the homogeneous complexes have exhibited satisfactory water oxidation activity, they are seldom applied in real solar water oxidation systems, mostly due to their poor photochemical stability. Comparatively, heterogeneous WOCs are robust and thus frequently supported on the surface of semiconductors for solar water oxidation.^19,20^

Nocera's group reported an *in situ* electrochemical synthesis of cobalt phosphate films on an indium tin oxide anode, which can oxidize water well under a neutral pH environment.^21^ Since then, the development of cobalt-based WOCs as well as their application in the fabrication of artificial photosynthesis devices has gained considerable attention. For example, Frei *et al.* synthesized ∼25 nm Co_3_O_4_ nanoclusters supported inside mesoporous silica (SBA-15 and KIT-6), and found that smaller Co_3_O_4_ clusters showed higher water oxidation activity.^22^ The surface of g-C_3_N_4_ modified with layered Co(OH)_2_ can not only accelerate the interface transfer rate of charge carriers, but also reduce the excessive energy barrier for O–O formation, thus leading to enhanced reaction kinetics for photocatalytic water oxidation.^23^ CoO_*x*_ as O_2_-evolving cocatalysts supported on the surface of a LaTiO_2_N photocatalyst also showed remarkable promotion of water oxidation performance under visible light irradiation.^19,24^ However, the size of CoO_*x*_ nanoparticles reported in most of the previous literature is usually larger than 5 nm, with a catalytic activity that is at least 2 orders of magnitude lower than those of homogeneous Co-based catalysts.^10^

Recently, many heterogeneous catalysts with single atoms or nanocluster structures have exhibited significantly enhanced catalytic activities with respect to conventional bulk catalysts.^25,26^ Inspired by this, one possible strategy to obtain highly active heterogeneous Co-based WOC is to reduce further the size of the cobalt oxide. However, the reduction of particle size is generally accompanied by an enhancement of surface energy, causing aggregation and instability of the WOC. Thus, further efforts on how to stabilize the ultrasmall nanoparticles should be considered. To address this, loading of ultrasmall nanoparticles onto the surface of a solid support has been known as effective way to stabilize nanoparticles.^10^ In addition, as for the application of WOC in photo(electro)catalytic water splitting, another design consideration is that the support should possess good conductivity for efficient carrier transfer. Based on these findings, the synthesis of ultrasmall Co-based WOCs anchored on the surface of a conductor is highly desirable. Graphite not only possesses good mechanical stability, but also exhibits excellent conductivity, and has been used widely in the field of solar cell and electrochemical water splitting.^27–29^ Accordingly, graphite is expected to be a good support to stabilize ultrasmall Co-based nanoparticles for further solar water splitting.

Herein, we report functionalized graphite-anchored CoO_*x*_ nanoparticles with an average size of sub-2 nm to exhibit unexpectedly efficient heterogeneous water oxidation activity. The sub-2 nm CoO_*x*_ nanoparticles were hydrothermally synthesized by anchoring them on the surface of phenylsulfonic acid-functionalized graphite. The TOF value of water oxidation on the optimized CoO_*x*_@G-Ph-SN sample can reach as high as 1.2 s^–1^, over two orders of magnitude higher than most heterogeneous transition metal oxides. In addition, the direct coupling of CoO_*x*_@G-Ph-SN with an Fe_2_O_3_ photoanode demonstrates its good photochemical stability under an artificial photosynthesis environment.

## Experimental

### Materials and reagents

All chemicals were analytical grade and used as purchased without further purification. Solutions were prepared using high purity water (Millipore Milli-Q purification system, resistivity > 18 MΩ cm). The fluorine-doped tin oxide (FTO) conductive glass was purchased from Nippon Sheet Glass Company (Japan) and was ultrasonically cleaned with acetone, ethanol and deionized water for 20 min each in sequence prior to use.

### Synthesis of samples

**Scheme 1:**
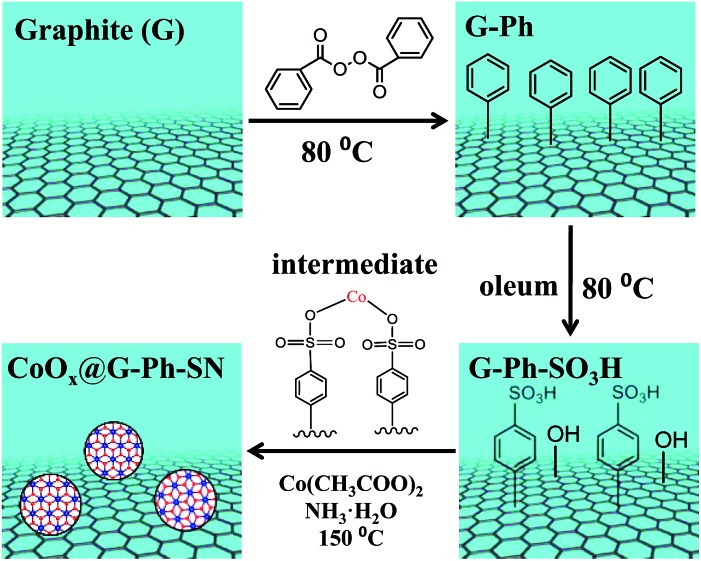
Schematic illustration of the synthesis of CoO_*x*_ nanoparticles anchored on sulfonated graphite (CoO_*x*_@G-Ph-SN).

As described in Scheme 1, the synthesis of the CoO_*x*_@G-Ph-SN sample mainly involves three steps: (i) immobilization of phenyl on the surface of graphite (G-Ph); (ii) sulfonation of phenyl (G-Ph-SO_3_H); and (iii) hydrothermal synthesis of CoO_*x*_ anchored on the surface of phenylsulfonic acid functionalized graphite (CoO_*x*_@G-Ph-SN). The first two steps were achieved by referring to previous work.^30^ As for the first step on immobilization of phenyl on the surface of graphite, typically, the dark gray graphite powder (0.5 g, 41.6 mmol of carbon) was dispersed in benzene (400 mL) in a 500 mL three necked round-bottom flask equipped with a magnetic stir bar. The contents were then stirred vigorously after benzoyl peroxide (10.1 g, 41.6 mmol) was added. The mixture was then heated at 80 °C for 12 h with continuous vigorous stirring. After cooling down, the contents of the flask were centrifuged and washed with ethanol for four times. The black solid was dried at 60 °C overnight, which was nominated as G-Ph.

As for the second step on phenylsulfonic acid functionalized graphite, a typical experimental process was as follows: phenylated graphite (G-Ph) (200 mg) was dispersed in oleum (70 mL, H_2_SO_4_, 25% as free SO_3_), and heated at 80 °C for 5 h to yield phenyl sulfonated graphite. After cooling down, 300 g of ice block was then carefully added into the suspension. The mixture was then centrifuged and washed with water several times until the pH value of the filtrate reached ∼7. The obtained solid was dried at 60 °C overnight, which was nominated as G-Ph-SO_3_H. For comparison, the pristine graphite was also treated with oleum in a similar process with the synthesis of G-Ph-SO_3_H, which was nominated as G–O.

As for the third step, the hydrothermal anchoring of CoO_*x*_ on the functionalized graphite (G-Ph-SO_3_H), typically 13.2 mg of Co(CH_3_COO)_2_·4H_2_O and 150 mg of G-Ph-SO_3_H were mixed in 20.0 mL of ethanol solution containing 50 µL of deionized water. After about 10 min stirring, 75 µL of 28% ammonia were added and mixed for another 10 min. Afterwards, the mixture was transferred into a 30 mL Teflon autoclave and heated at 150 °C for 2 h. After heat treatment, the autoclave was cooled to room temperature, and the product was washed with deionized water for more than three times. The final product was dried at 60 °C overnight, which was nominated as CoO_*x*_@G-Ph-SN.

In this work, the immobilization of phenyl and sulfonation of phenyl were confirmed by FTIR (ESI Fig. S1†), UV-Vis (ESI Fig. S2†) and XPS (ESI Table S1†), and the possible formation of cobalt benzenesulfonate intermediate can be verified by UV/Vis diffuse reflectance spectra (ESI Fig. S2 and S3†).^31^ As for discussion and comparison, Co_3_O_4_ nanoparticles (Co_3_O_4_-nano), CoO_*x*_@G–O and CoO_*x*_@graphite nanocomposites were similarly synthesized by following the above hydrothermal process (the third step) except that the former was free of G-Ph-SO_3_H, the middle employed G–O to substitute G-Ph-SO_3_H, and the latter employed graphite to substitute G-Ph-SO_3_H. Commercial Co_3_O_4_ was also employed for comparison.

### Preparation of photoanodes

The preparation of the nanoporous BiVO_4_ photoanode was as described in previous work.^32^ Typically, F-doped SnO_2_ (FTO) substrates were first electrochemically deposited with BiOI, and then a dimethylsulfoxide (DMSO) solution of vanadyl acetylacetonate [VO(acac)_2_] was dropped onto their surface and heated in air at 450 °C for 2 hours to obtain BiVO_4_ photoanodes. Afterwards, a calculated amount of pre-dispersed CoO_*x*_@G-Ph-SN ethanol solution was dropped onto the BiVO_4_ photoanode, which was then heated in air at 80 °C for 1 h to obtain the CoO_*x*_@G-Ph-SN/BiVO_4_ photoanode.

As for preparation of Fe_2_O_3_ photoanodes, Fe_2_O_3_ was deposited on an FTO substrate electrode using a modified chemical bath deposition method reported elsewhere.^33^ Afterwards, a calculated amount of pre-dispersed CoO_*x*_@G-Ph-SN ethanol solution was dropped onto the surface of the Fe_2_O_3_ photoanode, which was further heated in air at 65 °C for 10 min to produce the CoO_*x*_@G-Ph-SN/Fe_2_O_3_ photoanode.

### Characterizations of samples

The as-prepared samples were characterized by X-ray powder diffraction (XRD) on a Rigaku D/Max-2500/PC powder diffractometer. The sample powder was scanned using Cu Kα radiation with an operating voltage of 40 kV and current of 200 mA. A scan rate of 5° min^–1^ was applied to record the patterns in the range of 10–80°. Transmission electron microscope (TEM) images were observed by a Hitachi HT7700. High resolution TEM (HRTEM) images were recorded on a JEM-2100 transmission electron microscope (Tokyo, Japan) at 200 kV. The loading amount of cobalt oxide in the catalyst was determined using an inductively coupled plasma atomic emission spectrometer (ICP-AES) on a Shimadzu ICPS-8100. Prior to ICP-AES measurement, the supported cobalt oxide was dissolved in aqua regia. FT-IR spectra were obtained using a Varian 3100 FTIR spectrophotometer in DRIFT mode (diffuse reflectance infrared Fourier transform). The spectra were collected in the wavenumber range from 3900–400 cm^–1^ with 2 cm^–1^ resolution (average of 32 scans). The valence state of the cobalt oxide cluster was determined using XPS recorded on a Thermo ESCALAB 250Xi. The X-ray source selected was a monochromatized Al Kα source (15 kV, 10.8 mA). Region scans were collected using a 20 eV pass energy. Peak positions were calibrated relative to the C 1s peak position at 284.6 eV.

### Tests of chemical water oxidation

Typically, a calculated amount of 0.03–0.3 g L^–1^ CoO_*x*_@G-Ph-SN was added to a borate buffer solution (pH 9.0, 3.0 mL) containing Na_2_S_2_O_8_ (5.0 mM) and [Ru(bpy)_3_](ClO_4_)_2_ (1.0 mM). After 5 min stirring, the mixture was then irradiated with a halogen lamp through a coloured filter glass transmitting *λ* > 420 nm at room temperature. The photon flux of the incident light was determined to be 1.28 × 10^–7^ mol cm^–2^ s^–1^ using an EKO LS-100 spectroradiometer. Evolved oxygen gas was monitored by using a Clark-type electrode (Strathkelvin SI130 UK). The turnover frequency (TOF) was calculated by using the initial constant O_2_ evolution rate and assuming all the cobalt atoms were active sites. The contents of cobalt on typical samples were analyzed by ICP-AES measurement, which are 1.2 wt% and 1.29 wt% for CoO_*x*_@graphite and CoO_*x*_@G-Ph-SN, respectively.

### Tests of electrochemical water oxidation

The electrochemical water oxidation performances of all the cobalt-based electrodes were tested in a conventional three-electrode electrochemical cell with a platinum plate as the auxiliary electrode and a saturated calomel electrode (SCE, saturated KCl) as the reference electrode. A 1 M NaOH aqueous solution was used as the electrolyte with a pH measured at *ca.* 13.6. The scanning rate was 10 mV s^–1^. All potentials measured were calibrated to RHE using the following equation: *E*(RHE) = *E*(SCE) + 0.241*V* + 0.0591pH.

### Photoelectrochemical (PEC) measurements

The PEC tests were conducted in a three electrode system under simulated AM 1.5G solar light irradiation (100 mW cm^–2^, Newport Sol3A, Class AAA Solar simulator) or a 300 W xenon lamp (PLS-SXE300C, Perfectlight Company). The fabricated electrode, a platinum electrode, and a SCE were used as the working, counter and reference electrodes, respectively. 0.5 M lithium borate buffered solution (pH 9) or 1 M NaOH aqueous solution (pH = 13.6) was used as an electrolyte after saturation with Ar gas for 30 min. The photocurrent was measured by linear sweep voltammetry with a scan rate of 10 mV s^–1^. The light irradiation came from the front side of the electrodes in all cases.

### Kelvin probe force microscopy measurements

Kelvin Probe Force Microscopy (KPFM), which measures the contact potential difference between the probe and sample, was employed for potential imaging.^34^ An amplitude-modulated mode of the KPFM in lift mode was used. For each scan, a SCM-PIT Pt/Ir-coated conductive probe passes over the surface twice. On the first pass, a feedback loop controls the sample height to acquire the topography and phase signal of the sample. On the second pass, the cantilever is held at 100 nm of tip–sample distance to record the surface potential. The scan rate is 0.5 Hz.

## Results and discussion

**Fig. 1:**
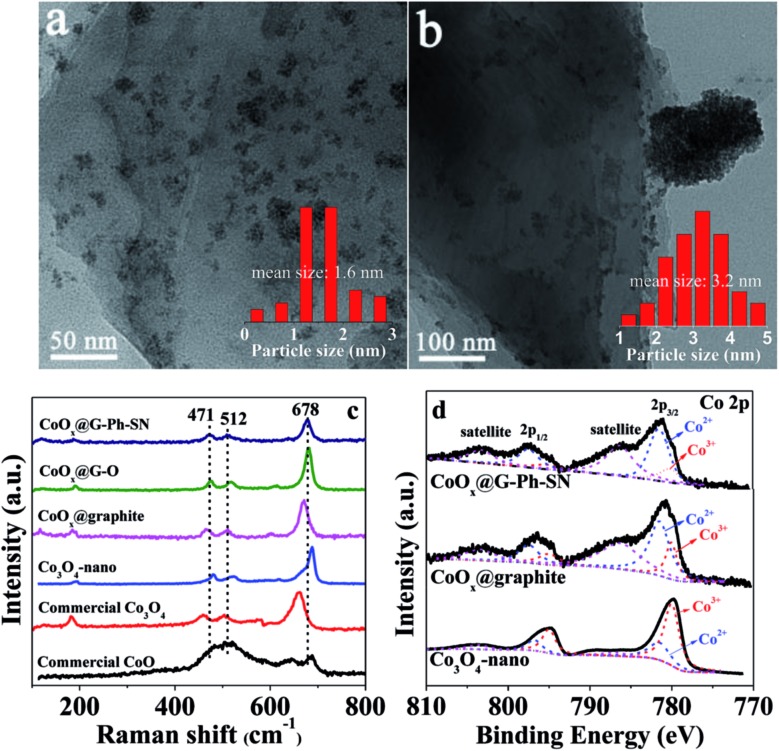
Representative TEM images of typical samples: (a) CoO_*x*_@G-Ph-SN, (b) CoO_*x*_@graphite; and their Raman (c) and XPS (d) spectra.

As seen in the TEM images of Fig. 1a and b, the CoO_*x*_ nanoparticles on the surface of CoO_*x*_@G-Ph-SN (Fig. 1a) are more homogeneously dispersed compared to the CoO_*x*_@graphite (Fig. 1b), CoO_*x*_@G–O (Fig. S4†) and Co_3_O_4_-nano (Fig. S4†); based on which their average sizes are calculated to be 1.6, 3.2, 2.5 and 5.3 nm, respectively. This demonstrates that compared to the synthesis of Co_3_O_4_-nano, the addition of graphite can inhibit the growth of CoO_*x*_ nanoparticles (CoO_*x*_@graphite) to a certain extent, but its aggregation (Fig. 1b) is clearly observed because of the hydrophobic surface feature of graphite itself. However, the surface of graphite after the functionalization of phenylsulfonic acid is changed from hydrophobic to hydrophilic according to the measurement of its contact angle (Fig. S5†). A hydrophilic surface is generally favorable for the homogeneous dispersion of CoO_*x*_ nanoparticles.^19^ In addition, the phenylsulfonic group is expected to complex with Co^2+^ according to comparison of the UV-Vis absorption results on typical samples (Fig. S2†), which should be another factor inhibiting its growth kinetics, causing the reduction of particle size. As an extended discussion, we also synthesized the oleum modified graphite for the loading of CoO_*x*_ nanoparticles. Similarly, the surface of graphite after treatment by oleum changes from hydrophobic to hydrophilic according to the measurement of its contact angle (Fig. S5†), and after loading the same amount of CoO_*x*_ on the oleum treated graphite, the dispersion of CoO_*x*_ can be improved and the particle size can be reduced compared with those on the pristine graphite (Fig. S4†). However, the average particle size of CoO_*x*_ on the oleum treated graphite is 2.5 nm, which is a little larger than that (*ca.* 1.6 nm) loaded on the surface of GE-Ph-SO_3_H. This means that surface wettability modification caused by oleum treatment can improve the dispersion and decrease the size of CoO_*x*_ to a certain extent, but its influence is not as obvious as that caused by the surface phenyl-sulfonation modification. This difference may lie in the complex effect of Co^2+^ ions with phenylsulfonic groups, as this may change the number of crystal nuclei as well as the concentration of Co^2+^ ions remaining in the solution, leading to different growth kinetics.

The states of the cobalt species existing on the samples were analyzed by Raman and XPS spectra. As seen in Fig. 1c, three prominent Raman peaks, located at 678, 512, and 471 cm^–1^ can be observed for the CoO_*x*_@G-Ph-SN, CoO_*x*_@G–O and CoO_*x*_@graphite samples, which are not consistent with the pure CoO phase, Co_3_O_4_ phase, or nano-Co_3_O_4_ phase.^35^ This demonstrates that the grafted cobalt species differ from the single phase of CoO or Co_3_O_4_. The valence state of CoO_*x*_ in CoO_*x*_@G-Ph-SN is further investigated by Co 2p XPS (Fig. 1d), in which two prominent shake-up satellite peaks, indicative of Co^2+^ ions,^36^ are clearly observed, and the Co 2p_1/2_–Co 2p_3/2_ energy separation is approximately 16.0 eV. Based on our fitted curves of Co 2p_3/2_ peak, the surface atomic ratios of Co^3+^/Co^2+^ are calculated to be *ca.* 0.35, 0.38 and 2.0 for CoO_*x*_@G-Ph-SN, CoO_*x*_@graphite and Co_3_O_4_-nano, respectively. The high concentration of Co^2+^ in CoO_*x*_@graphite and CoO_*x*_@G-Ph-SN can be further verified by EPR spectra (Fig. S6†).

To characterize further the CoO_*x*_ particles on graphite, we carried out XRD and HRTEM measurements. No obvious XRD peaks assigned to the CoO_*x*_ can be observed even for the sample with a CoO_*x*_ loading content up to *ca.* 10.0 wt% (Fig. S7†). Based on the HRTEM image (Fig. S8†), the space distance of the CoO_*x*_ can be calculated to be 0.251 nm, which is not in accordance with those of CoO, Co(OH)_2_, CoOOH and Co_3_O_4_. Thus, the CoO_*x*_ phase in this work should be different from each of them. Based on the above analysis, we thus label the cobalt species in this work as CoO_*x*_ for simplicity. The surface element contents of CoO_*x*_@graphite, CoO_*x*_@G–O and CoO_*x*_@G-Ph-SN samples were analyzed and are given Table S1.†

**Fig. 2:**
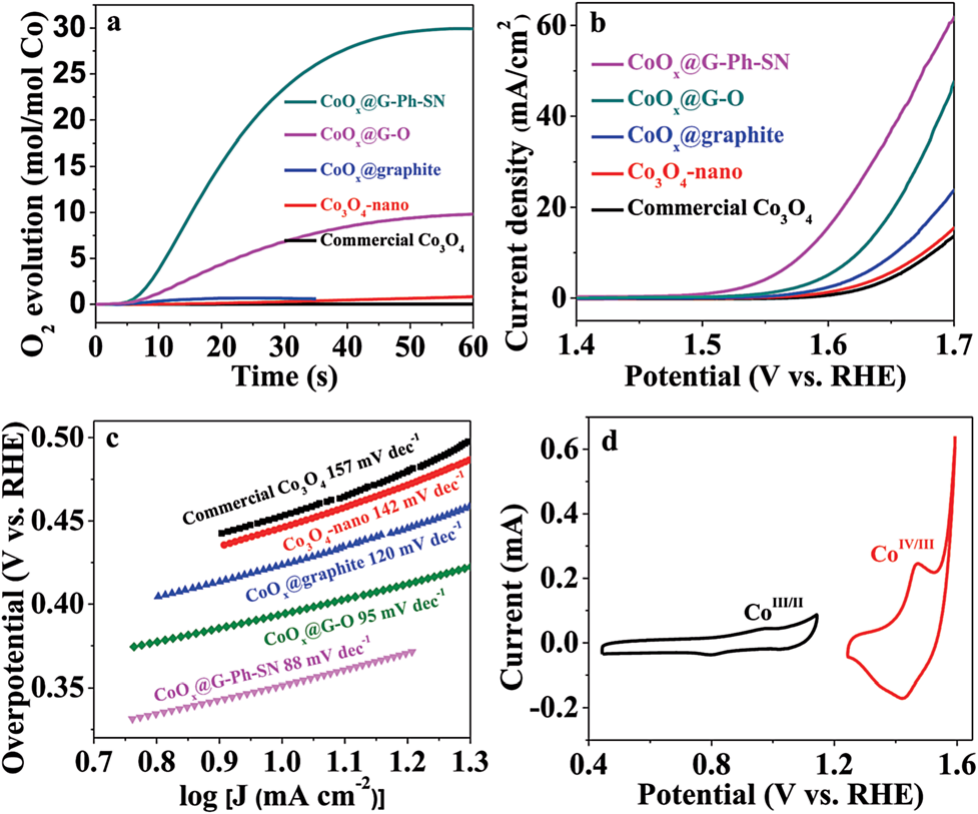
Photochemical (a) and electrochemical (b–d) water oxidation performances of typical samples. (a) Time profiles of light-driven O_2_ evolution catalyzed by various catalysts; (b) LSV curves of various catalysts in 1.0 M NaOH; (c) Tafel plots derived from (b). (d) Cyclic voltammograms (CVs) of CoO_*x*_@G-Ph-SN in 1.0 M NaOH at a scan rate of 10 mV s^–1^. Reaction conditions: 0.5 mg catalyst, 3 mL H_2_O, [Ru^II^(bpy)_3_]Cl_2_ (1.0 mM), and Na_2_S_2_O_8_ (5.0 mM) in borate buffer (80 mM, pH 9).

To evaluate the potential of the as-obtained CoO_*x*_@G-Ph-SN sample as a WOC, a visible-light-driven water oxidation system containing [Ru(bpy)_3_]Cl_2_ and Na_2_S_2_O_8_ in the presence of a borate-buffered solution was examined with oxygen detected by a Clark electrode. Fig. 2a gives their typical activity curves as a function of reaction time, based on which their TOF values of water oxidation are calculated. As a comparison, Co_3_O_4_-nano (TOF of 0.012 s^–1^) shows an obvious promotion of water oxidation activity compared to the commercial Co_3_O_4_ (TOF of 0.0013 s^–1^). The photocatalytic water oxidation activity can be enhanced by the CoO_*x*_@graphite sample, showing a TOF of 0.058 s^–1^. The photocatalytic water oxidation activity can be further enhanced by improving the dispersion of CoO_*x*_ and decreasing the particle size of CoO_*x*_. A TOF of 0.31 s^–1^ can be achieved over CoO_*x*_@G–O. Comparatively, the CoO_*x*_@G-Ph-SN sample shows the best TOF value of 1.2 s^–1^, an unexpectedly efficient water oxidation activity with respect to previously reported cobalt-based oxides (Table S2†). It should be pointed out that the TOF value is related to the light intensity (Fig. S9†). In addition, the water oxidation performances are normally affected by the surface reaction and charge separation processes, so factors beyond the size of CoO_*x*_ should have an effect on the activity; these probably include the mass transfer at the interface of the catalyst and aqueous solution, the structures of the active species and the the good conductivity of graphite *etc.*

The water oxidation performance was also evaluated by electrochemical water oxidation. Their linear sweep voltammetry (LSV) curves are depicted in Fig. 2b. The glassy carbon electrode (GCE) or graphite itself shows a negligible current and very high onset potential. Similarly, the water oxidation activity trend on typical electrodes can be described as follows: CoO_*x*_@G-Ph-SN > CoO_*x*_@G–O > CoO_*x*_@graphite > Co_3_O_4_-nano > commercial Co_3_O_4_, whose overpotential values at a current density of 10 mA cm^–2^ are 350 mV, 395 mV, 450 mV, 470 mV, and 475 mV, respectively. The excellent O_2_-evolving activity of the CoO_*x*_@G-Ph-SN composite was further confirmed by its much smaller Tafel slope (88 mV per decade) at lower overpotentials than that measured for CoO_*x*_@G–O (95 mV per decade), CoO_*x*_@graphite (120 mV per decade), Co_3_O_4_-nano (142 mV per decade), and commercial Co_3_O_4_ (157 mV per decade) (Fig. 2c). The cyclic voltammogram (CV) of CoO_*x*_@G-Ph-SN in 1.0 M NaOH solution at a scan rate of 10 mV s^–1^ (Fig. 2d) revealed two reversible reduction waves at *E*_1/2_ = 0.89 V and 1.44 V *versus* RHE, assigned to two sequentially occurring one-electron redox reactions involving Co^II^/Co^III^ and Co^III^/Co^IV^ couples, respectively.^37–39^

**Fig. 3:**
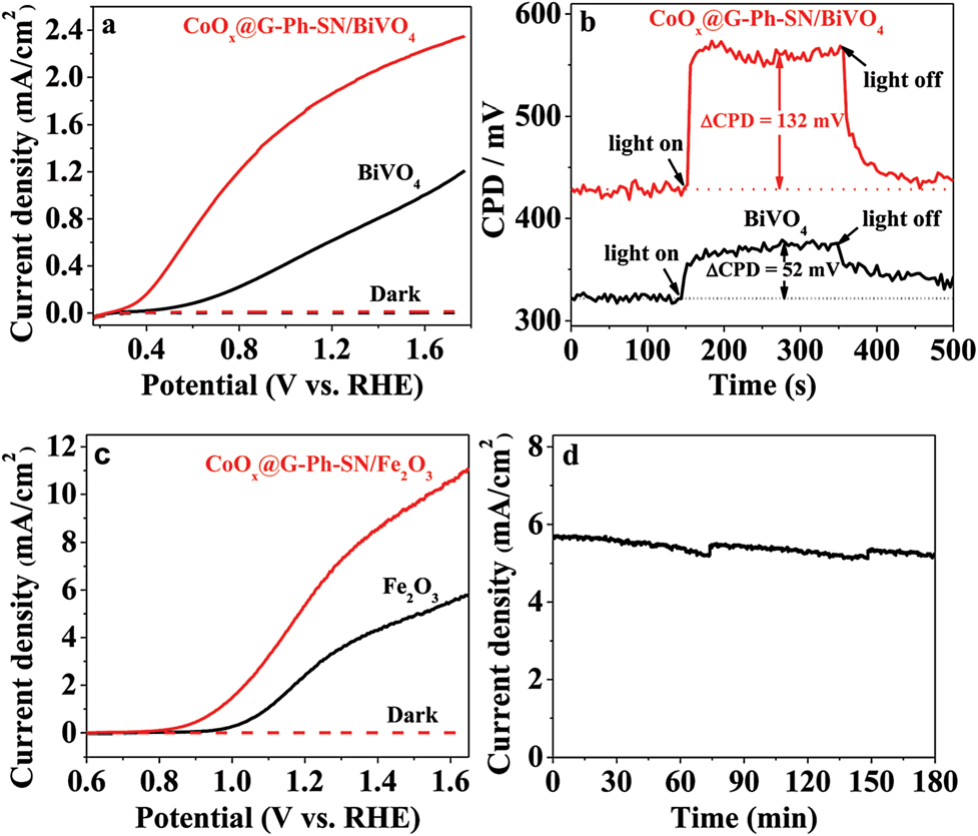
Comparison of the photoelectrochemical performances and characterization of BiVO_4_ and/or Fe_2_O_3_ photoanodes with and without loading of CoO_*x*_@G-Ph-SN. (a) LSV curves of BiVO_4_ photoanodes; 0.5 M lithium borate buffered solution (pH 9); 100 mW cm^–2^ AM1.5G; scan rate of 50 mV s^–1^. (b) Kelvin probe force microscopy of BiVO_4_ photoanode. (c) LSV curves of Fe_2_O_3_ photoanodes; 1.0 M KOH; 300 W xenon lamp; scan rate of 50 mV s^–1^. (d) Photoelectrochemical stability of CoO_*x*_@G-Ph-SN/Fe_2_O_3_ photoanode at 1.23 V *vs.* RHE. Electrolyte: 1.0 M KOH; light source: 300 W xenon lamp.

Encouraged by the extraordinary water oxidation activity on the CoO_*x*_@G-Ph-SN sample, we thus further evaluated its potential use in a practical artificial photosynthesis system. As an initial attempt, we loaded the CoO_*x*_@G-Ph-SN onto the surface of a BiVO_4_ electrode for photoelectrochemical water oxidation. Here the CoO_*x*_@G-Ph-SN WOC can be considered as a cocatalyst of the BiVO_4_ photoanode for water oxidation.^40^ Fig. 3a gives the typical linear sweep voltammetry (LSV) curves of BiVO_4_ photoanodes with and without loading of CoO_*x*_@G-Ph-SN, in which loading of CoO_*x*_@G-Ph-SN not only obviously promotes the current of the BiVO_4_ photoanode, but also causes a negative shift of the onset potential. This clearly demonstrates that the CoO_*x*_@G-Ph-SN is not only more active for water oxidation than BiVO_4_ itself, but also efficient for the extraction of holes reaching the surface of BiVO_4_ for efficient transfer as well as promoted charge separation. The efficient transfer of photo-generated carriers between BiVO_4_ and CoO_*x*_@G-Ph-SN is confirmed by analysis of KPFM. As given in Fig. 3b, the steady state contact potential difference (ΔCPD) of the BiVO_4_ photo-electrode with and without light irradiation is more significantly increased after the loading of CoO_*x*_@G-Ph-SN.

The effectiveness of CoO_*x*_@G-Ph-SN as a cocatalyst of water oxidation to promote the performance of artificial photocatalysts can be further revealed by loading it on the surface of another robust Fe_2_O_3_ photoanode. As revealed by the LSV curves of Fig. 3c, the loading of CoO_*x*_@G-Ph-SN on the surface of Fe_2_O_3_ photoanode not only negatively shifts the onset potential of the photocurrent by *ca.* 150 mV, but also promotes the photocurrent at 1.23 eV (*vs.* RHE) by about 2.1 times. It is worth noting that the photocurrent of the CoO_*x*_@G-Ph-SN/Fe_2_O_3_ electrode at 1.23 V *vs.* RHE can be maintained by over 90% after 3 h irradiation, indicating the good photochemical stability of the CoO_*x*_@G-Ph-SN catalyst as a water oxidation cocatalyst in artificial photosynthesis. Together with the promotion of charge separation, we can reasonably deduce that CoO_*x*_@G-Ph-SN, with high activity and stability, is a promising WOC for artificial photosynthesis.

## Conclusions

In summary, ultrasmall CoO_*x*_ nanoparticles (around 1.6 nm) anchored on the surface of sulfonated graphite have been synthesized by a simple hydrothermal process. The success of the synthesis in reducing particle size mainly originates from the improvement of the surface hydrophilicity of graphite as well as its possible complex effect with Co^2+^ ions, leading to homogeneous dispersion and retarded growth. The as-obtained nanocomposite exhibits unexpected photochemical water oxidation activity with an optimal TOF of 1.2 s^–1^, at least two orders of magnitude higher than that of conventional cobalt-based oxides. Furthermore, the CoO_*x*_@G-Ph-SN can be grafted on the surface of artificial photocatalysts like BiVO_4_ and Fe_2_O_3_ for promoted photoelectrochemical water oxidation, demonstrating its bright future in constructing solar-to-chemical energy conversion systems.
