# Supplementary material for: CoOx nanoparticle anchored on sulfonated-graphite as efficient water oxidation catalyst
Source: Chem Sci. 2017 Jun 26;8(9):6111–6. doi: 10.1039/c7sc01756a (PMC5625584; doi:10.1039/c7sc01756a)
Supplement: Supplementary file 1 [file SC-009-C7SC01756A-s001.pdf]

Electronic Supplementary Material (ESI) for Chemical Science.
This journal is © The Royal Society of Chemistry 2017

Supporting Information

CoO_x Nanoparticle Anchored on Sulfonated-graphite as Efficient Water Oxidation Catalyst

Jingqi Guan, Chunmei Ding, Ruotian Chen, Baokun Huang, Xianwen Zhang, Fengtao Fan, Fuxiang Zhang* and Can Li*

State Key Laboratory of Catalysis, *i*ChEM, Dalian Institute of Chemical Physics, Chinese Academy of Sciences, Dalian National Laboratory for Clean Energy, Dalian, 116023, China. E-mail: fxzhang@dicp.ac.cn; canli@dicp.ac.cn

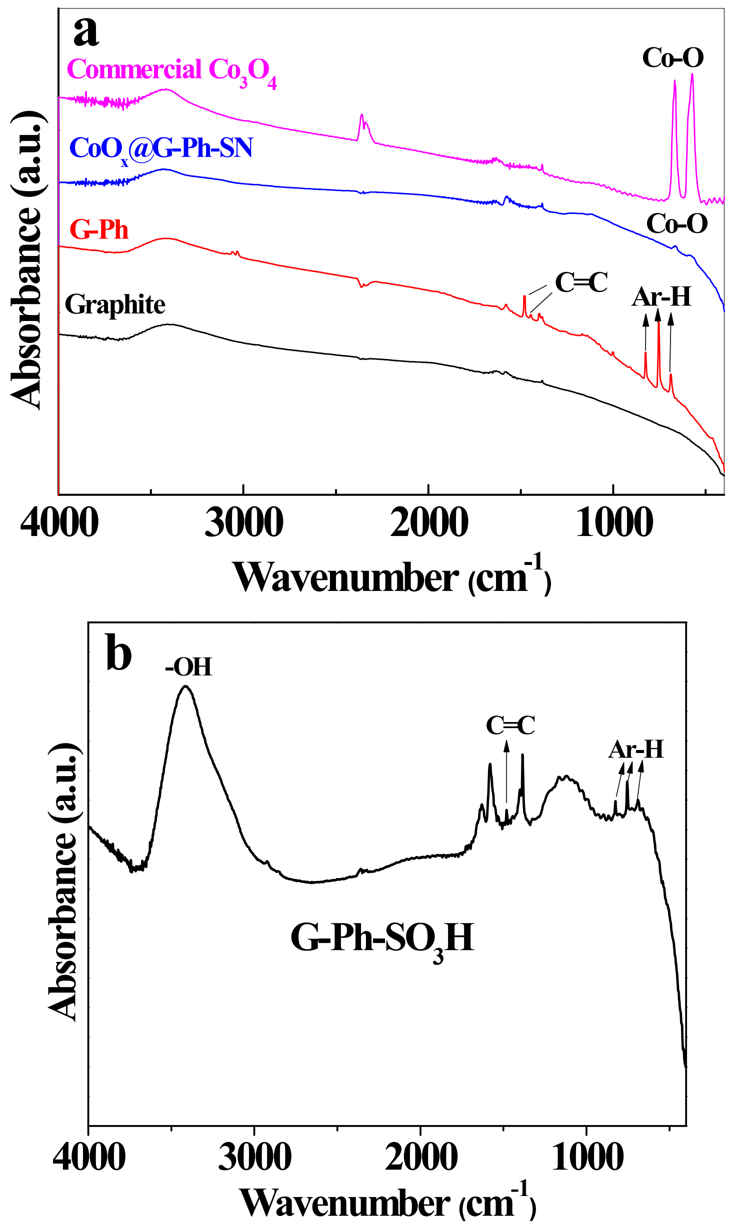

Fig. S1. FTIR spectra of (a) graphite, G-Ph, CoO_x@G-Ph-SN, and commercial Co_3O_4 and (b) G-Ph-SO_3H.

From Fig. S1a, the peaks at 687, 753 and 832 cm^{-1} assigned to the Ar-H vibrations, and peaks at 1455 and 1480 cm^{-1} due to the C=C vibrations can be clearly seen for the G-Ph sample, suggesting the successfully anchoring of phenyl on the surface of graphite. After further surface sulfonation, the peaks at 687, 753, 832, and

1480 cm^{-1} assigned to the Ar-H and C=C vibrations of phenyl groups for G-Ph-SO_3H can be still seen (Fig. S1b), although the intensities of these peaks are decreased. This demonstrates that part of phenyl groups fell off during the sulfonation process. However, it should be pointed out that the content of the sulfur element analyzed by XPS (see Table S1) is as large as 0.4 mol%, demonstrating the amount of phenylsulfonic groups on the surface of graphite is still large. After the hydrothermal treatment, the bands (571 and 674 cm^{-1}) related to Co-O bonds can be found, suggesting that the CoO_x nanoparticles have been well supported on the surface of functionalized graphite (G-Ph-SO_3H).

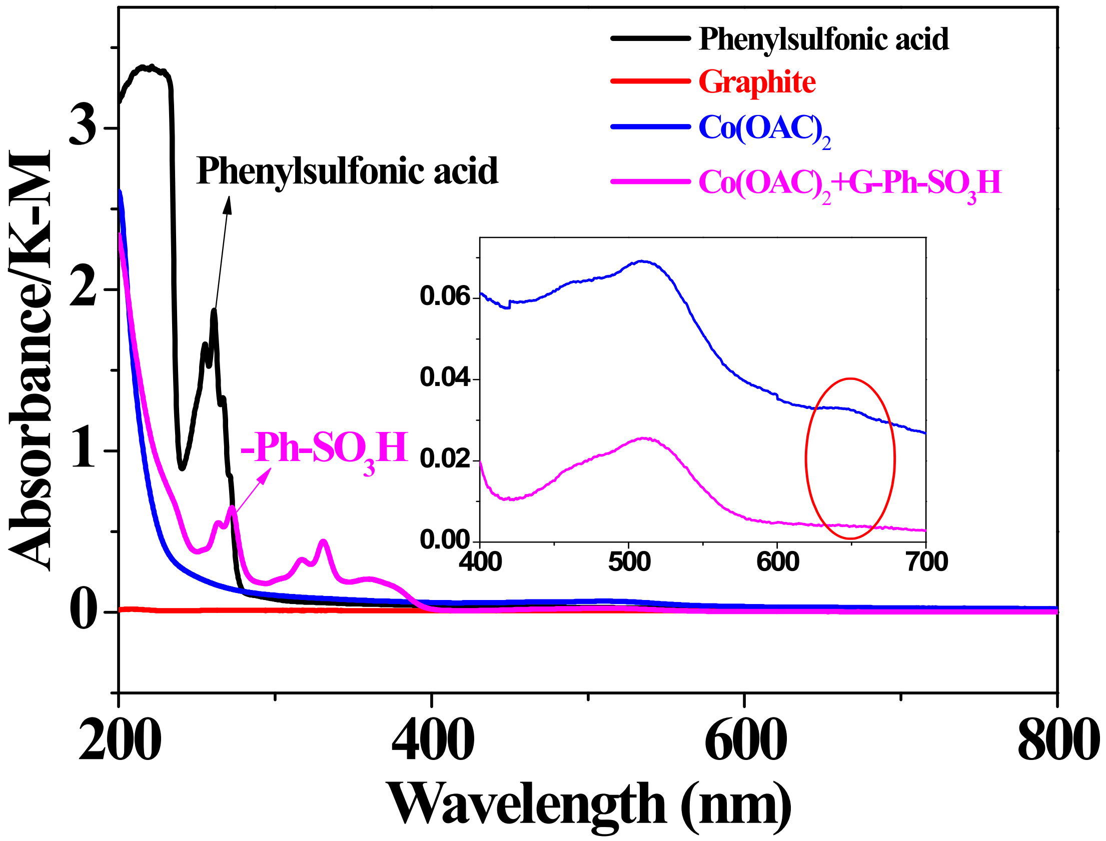

Fig. S2. UV/Vis diffuse reflectance spectra of various solutions: 100 mM phenylsulfonic acid (black), graphite (red), 5 mM cobaltous acetate (blue), and the mixture of 5 mM cobaltous acetate and G-Ph-SO_3H (pink).

From Fig. S2, the main UV-Vis absorption peak of the benzenesulfonate group shifts from 262 nm to 273 nm after anchoring it onto the surface of graphite, indicating the success of the synthesis. In addition, the UV-Vis absorption peak corresponding to the cobaltous acetate centred at ca. 644 nm disappears after adding G-Ph-SO_3H to the solution of cobaltous acetate, suggesting the the possible formation of cobalt benzenesulfonate intermediate as further confirmed by the followed Fig. S3.

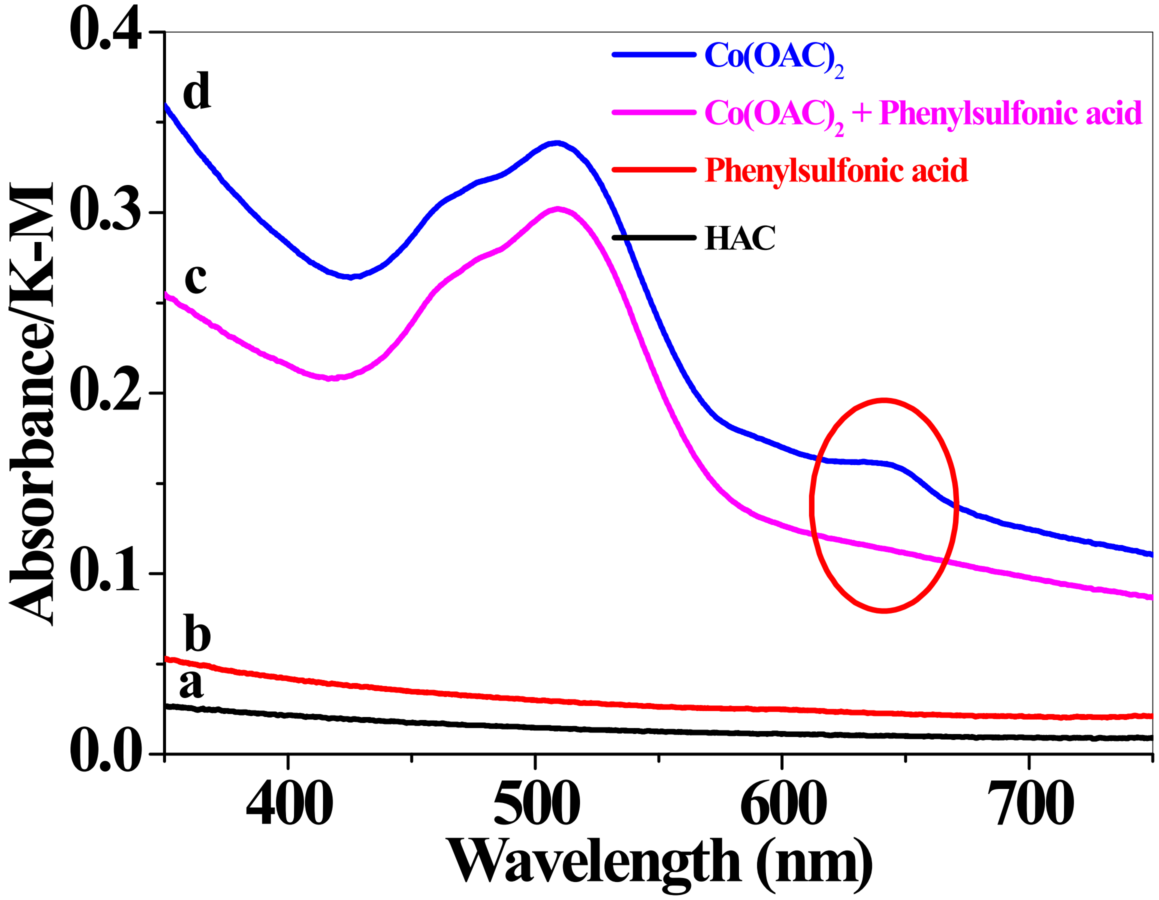

Fig. S3. UV/Vis diffuse reflectance spectra of various solutions: (a) 50 mM acetic acid, (b) 50 mM phenylsulfonic acid, (c) the isometric mixture of 50 mM cobaltous acetate and 100 mM phenylsulfonic acid, and (d) 25 mM cobaltous acetate.

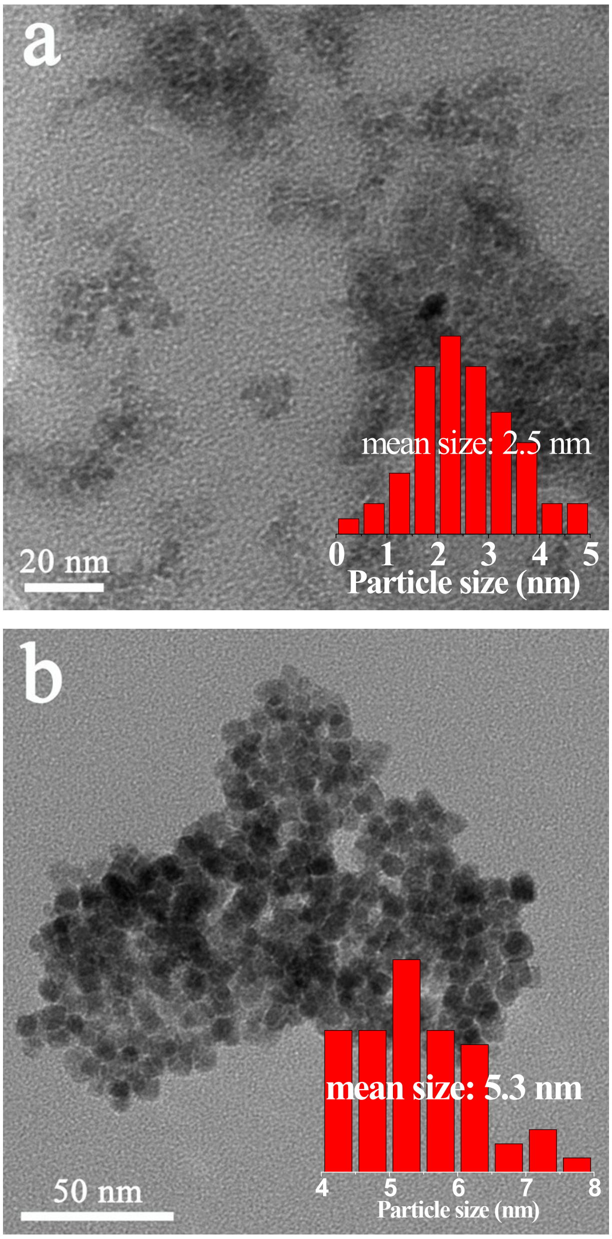

Fig. S4. TEM images and histogram of size distributions of CoO_x@G-O (a) and Co_3O_4-nano (b).

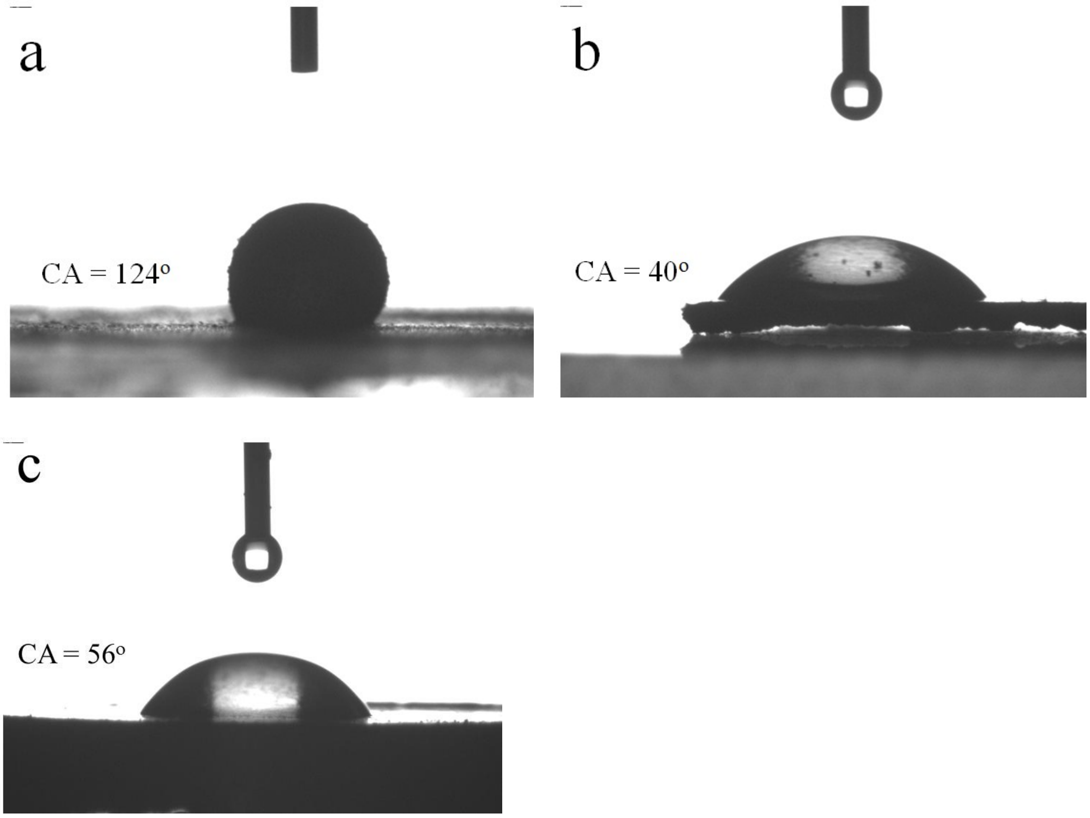

Fig. S5. Contact angles (CAs) of (a) graphite, (b) G-Ph-SO_3H and (c) G-O.

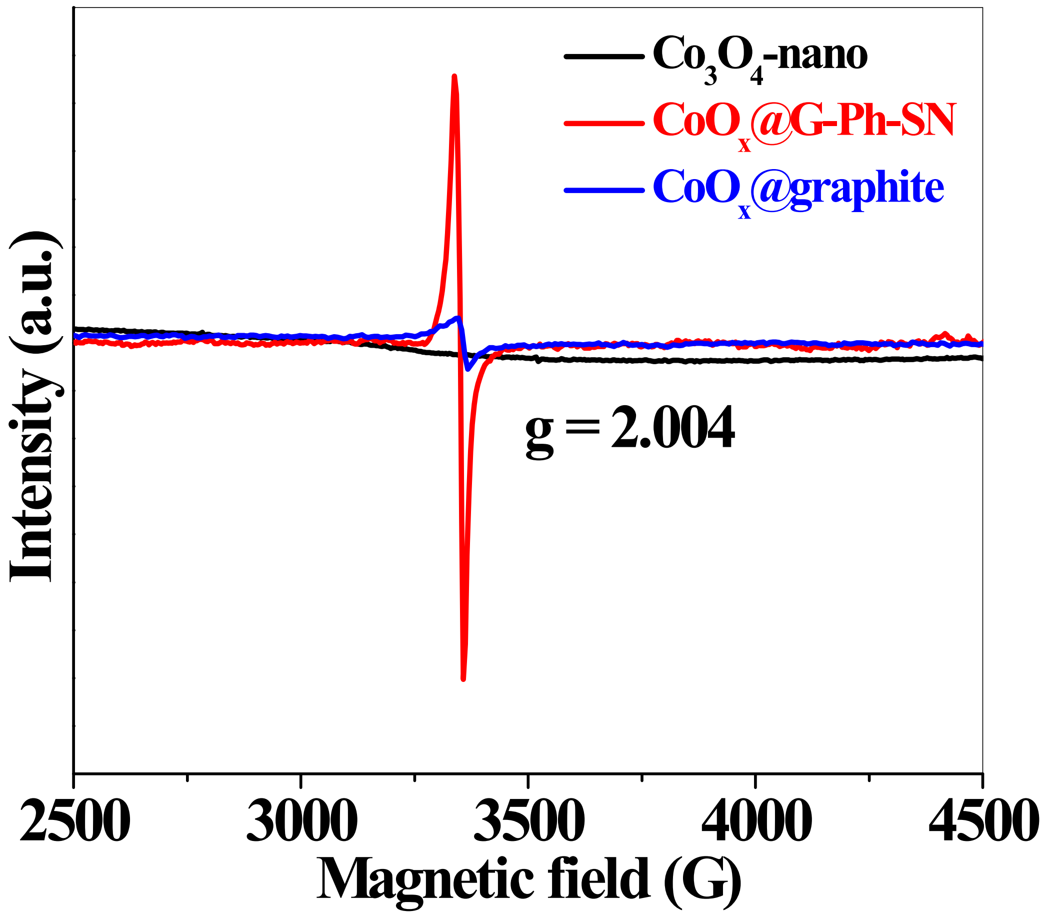

Fig. S6. EPR spectra of Co_3O_4-nano, CoO_x@graphite, and CoO_x@G-Ph-SN.

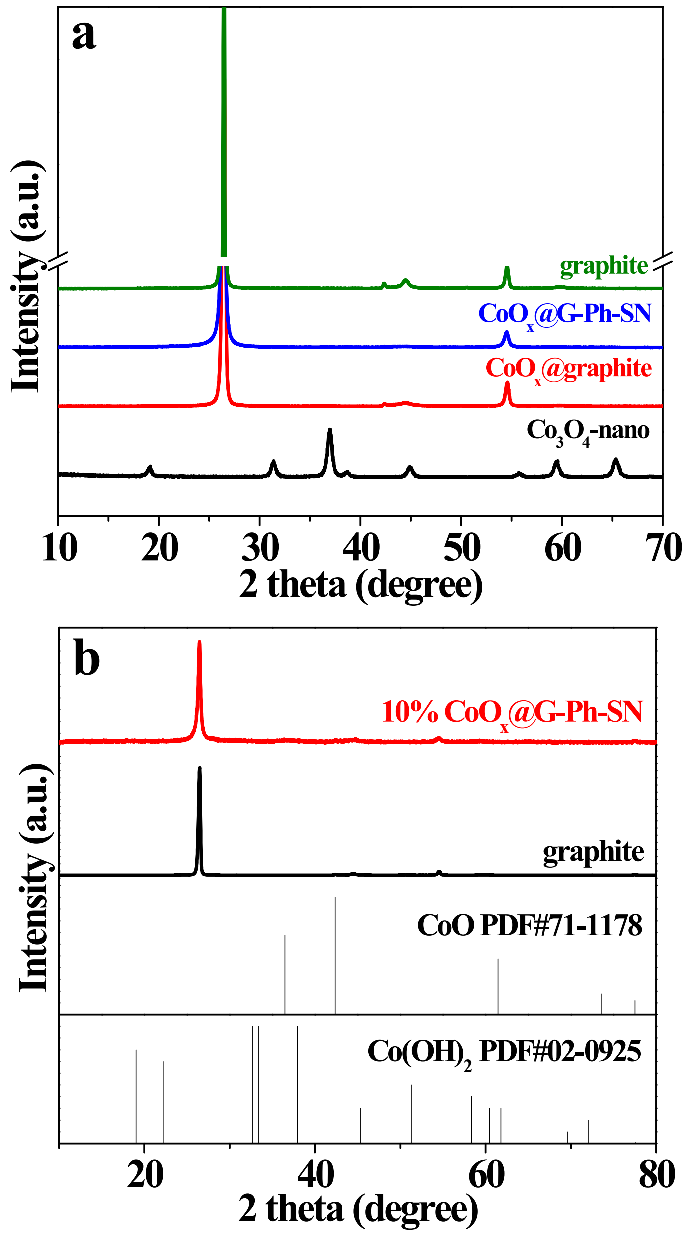

Fig. S7. XRD patterns of (a) graphite, CoO_x@G-Ph-SN, CoO_x@graphite and Co_3O_4-nano; and (b) graphite, 10% CoO_x@G-Ph-SN, CoO and $Co(OH)_2$.

10% CoO_x@G-Ph-SN was similarly synthesized by following the hydrothermal process of CoO_x@G-Ph-SN except that the initial $Co(CH_3COO)_2•4H_2O$ amount increased to 132 mg and the added ammonia amount increased to 750 µL.

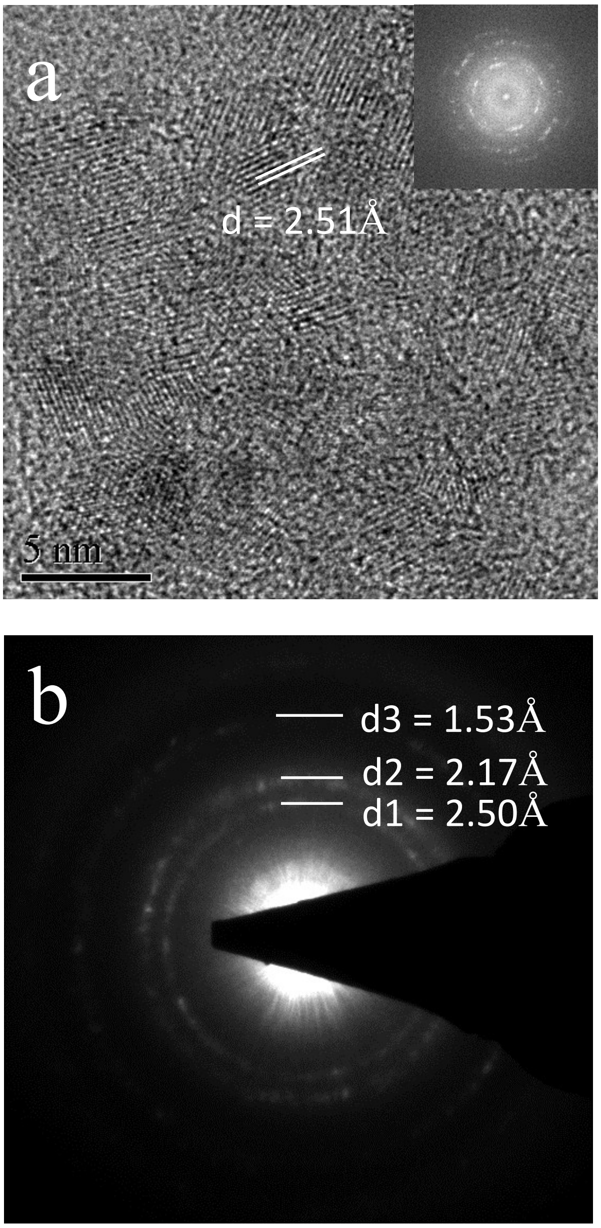

Fig. S8. HRTEM image of CoO_x@G-Ph-SN (a) and SAED pattern of CoO_x (b).

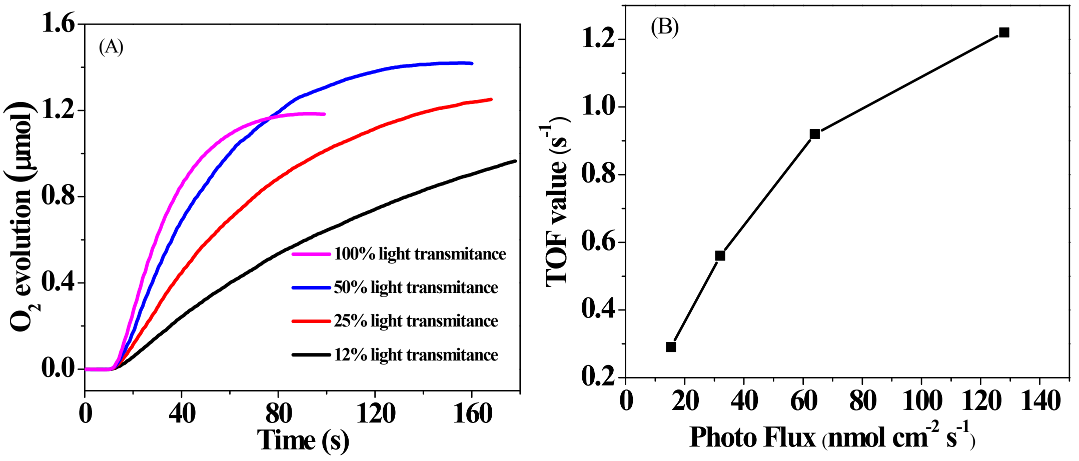

Fig. S9. Investigation of TOF value as a function of photo flux: (A) Time course of O_2 evolution; (B) Dependence of TOF value as the photo flux.

Reaction conditions: 0.15 mg catalyst, 3 mL H_2O, $[Ru^{II}(bpy)_3]Cl_2$ (1.0 mM), and $Na_2S_2O_8$ (5.0 mM) in borate buffer (80 mM, pH 9). As for the 100% light transmittance, the photo flux is 1.28×10^{-7} mol cm^{-2} s^{-1}, which was measured by an EKO LS-100 spectroradiometer. The light intensity was controlled by using neutral density filter (ZND0012, ZND0025 and ZND0050), which can keep spectrally even performance in VIS range.

As seen in Fig. S9, the TOF value is first linearly increased and then slowly increased with the increasing light intensity. The TOF value reaches a maximum under the 100% light transmittance and can not be increased even by using higher intensity light source to irradiate (e.g. 300 W xenon). It should be noted that the $Ru(bpy)_3{}^{2+}$ is not stable under very strong light irradiation (e.g. 300 W xenon).

Table S1 Surface elemental contents of CoO_x@graphite, CoO_x@G-O and CoO_x@G-Ph-SN analyzed by XPS

Catalysts	Surface composition (*mol.*%)				
	C	O	N	S	Co
CoO_x@graphite	96.8	2.9	-	-	0.3
CoO_x@G-O	93.1	6.4	0.1	0.1	0.3
CoO_x@G-Ph-SN	95.5	3.4	0.4	0.4	0.3

Table S2 Comparison of the catalytic performance of various Co-based water oxidation catalysts

Catalysts	Oxidant	TOF (s^{-1})	References
CoO_x@G-Ph-SN (~1.6 nm)	$Ru(bpy)_3^{3+}$	1.2	This work
Co_3O_4	$Ru(bpy)_3^{3+}$	$1.4*10^{-4}$	[1]
Nano Co_3O_4 (~ 6 nm)	$Ru(bpy)_3^{3+}$	$2.5*10^{-4}$	[2]
SBA-15/Co_3O_4	$Ru(bpy)_3^{3+}$	$3.4*10^{-4}$	[2]
SBA-15/Co_3O_4	Ce(IV)	$6.4*10^{-4}$	[3]
Mesoporous Co_3O_4	Ce(IV)	$\sim 2.2*10^{-3}$	[4]
Mesoporous Mg−Co_3O_4	Ce(IV)	$1*10^{-3}$	[4]
Co_3O_4/mesoporous silica	Ce(IV)	$\sim 4*10^{-4}$	[4]
$ZnCo_{1.0}O_y$	$Ru(bpy)_3^{3+}$	$3.2*10^{-3}$	[5]
$Co_4O_4(py)_4(Ac)_4$	$Ru(bpy)_3^{3+}$	0.02	[6]

References

1. A. Harriman, I. J. Pickering, J. M. Thomas and P. A. Christensen, *J. Chem. Soc., Faraday Trans.*, 1988, **84**, 2795.
2. S. Yusuf and F. Jiao, *ACS Catal.*, 2012, **2**, 2753.
3. F. Jiao and H. Frei, *Angew. Chem. Int. Ed.*, 2009, **48**, 1841.
4. J. Rosen, G. S. Hutchings and F. Jiao, *J. Am. Chem. Soc.*, 2013, **135**, 4516.
5. F. Rong, J. Zhao, P. Su, Y. Yao, M. Li, Q. Yang and C. Li, *J. Mater. Chem. A*, 2015, **3**, 4010.
6. N. S. McCool, D. M. Robinson, J. E. Sheats and G. C. Dismukes, *J. Am. Chem. Soc.*, 2011, **133**, 11446.
